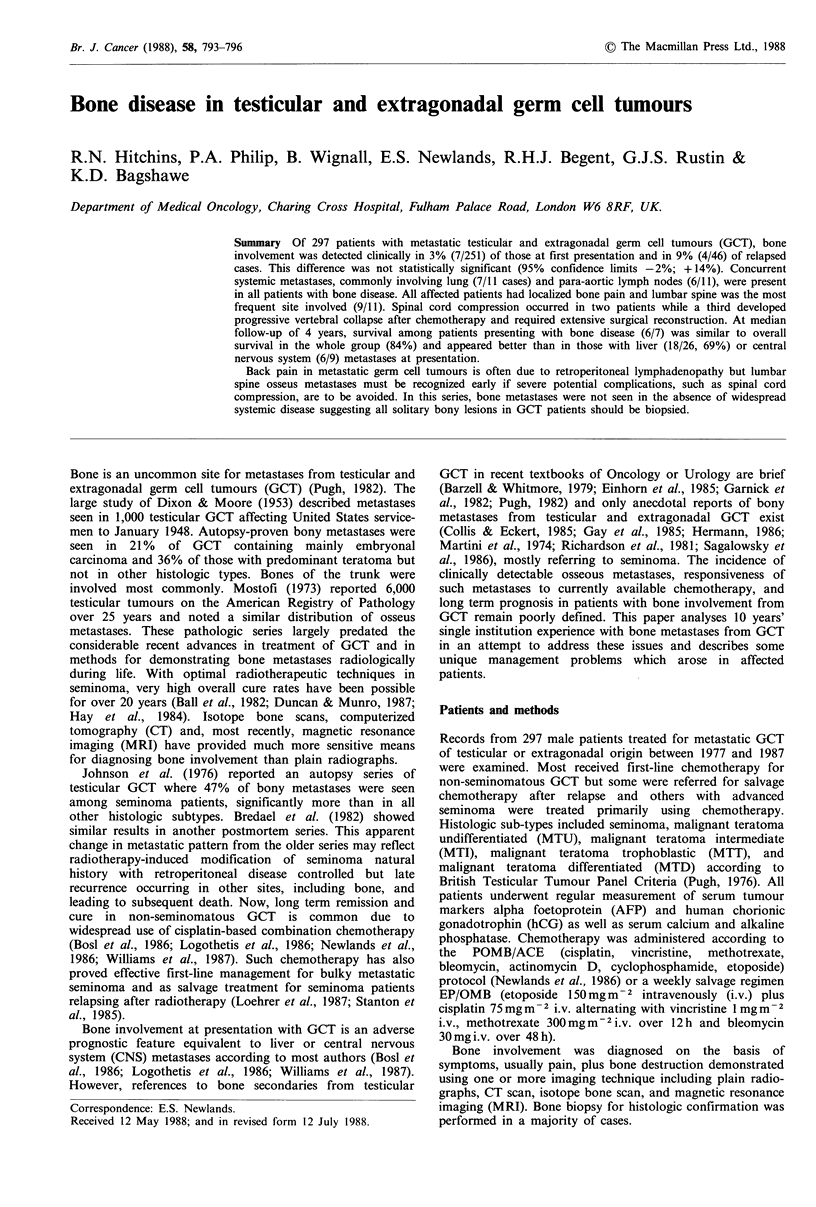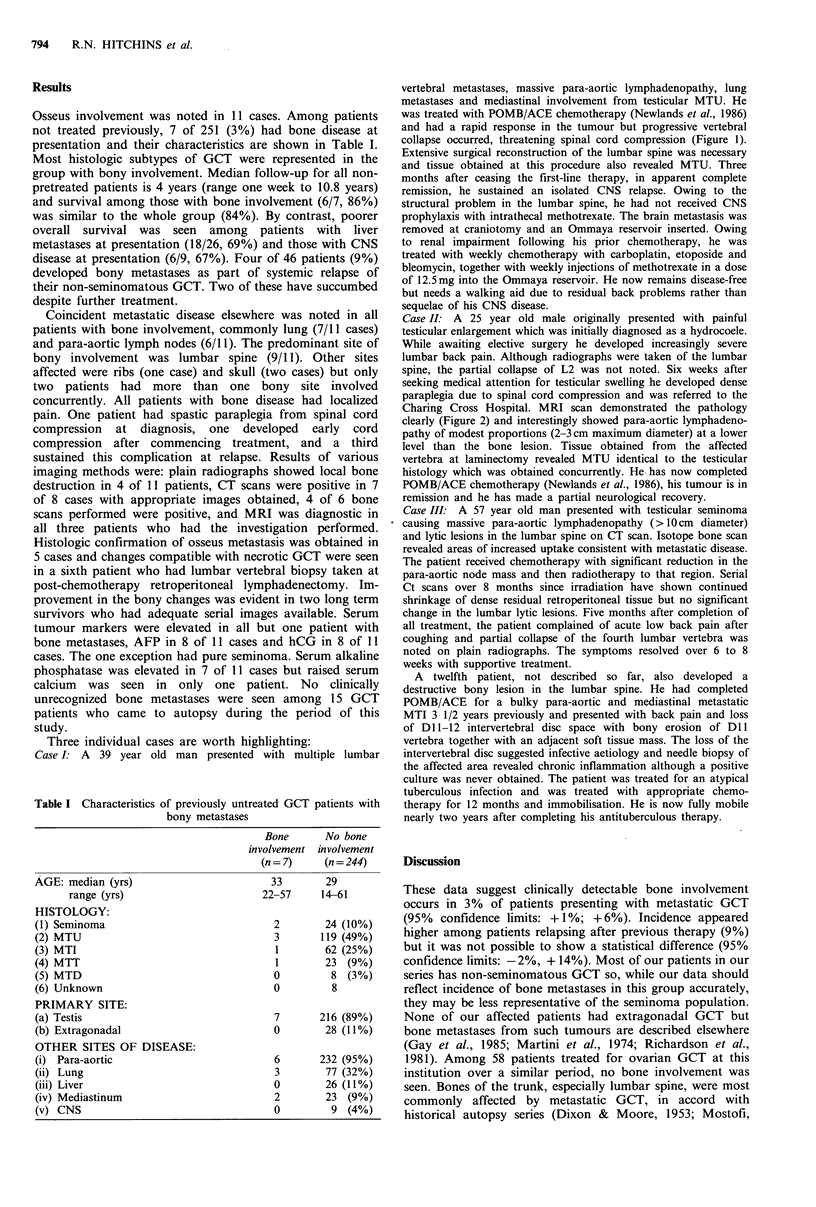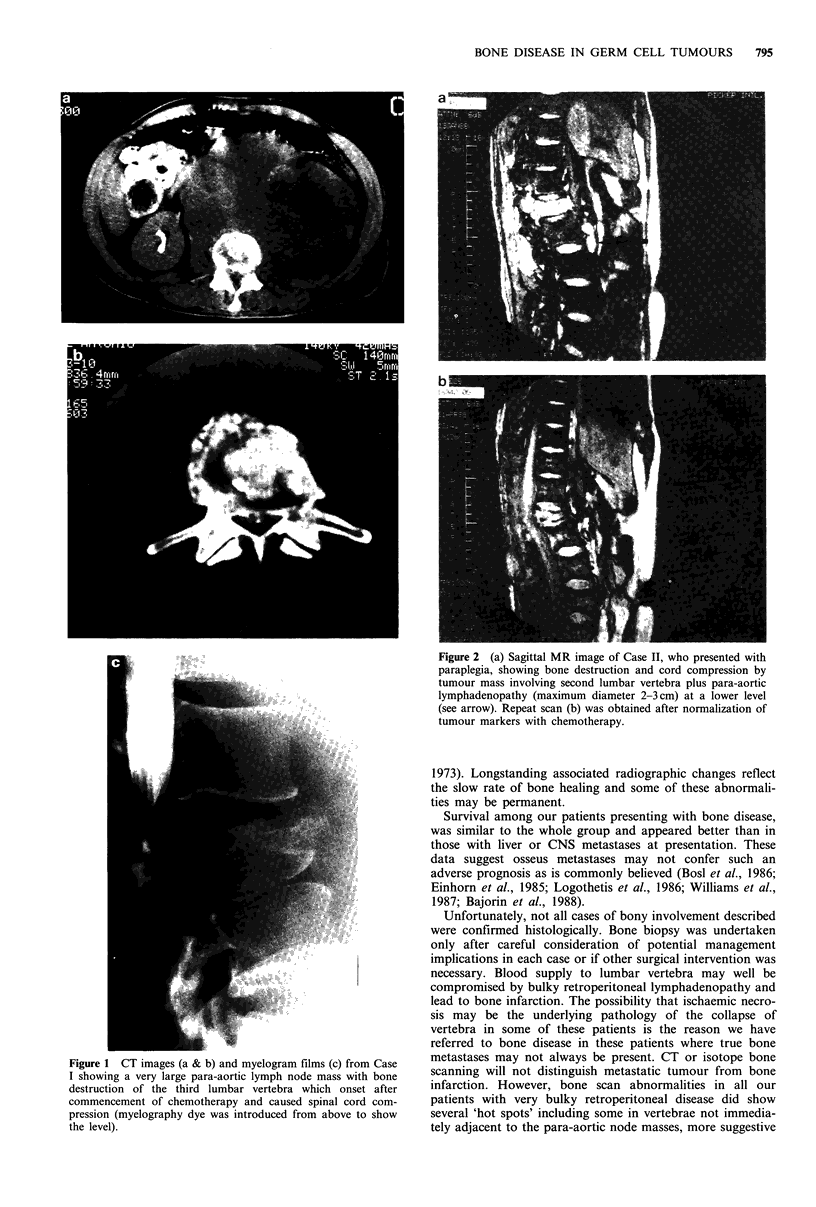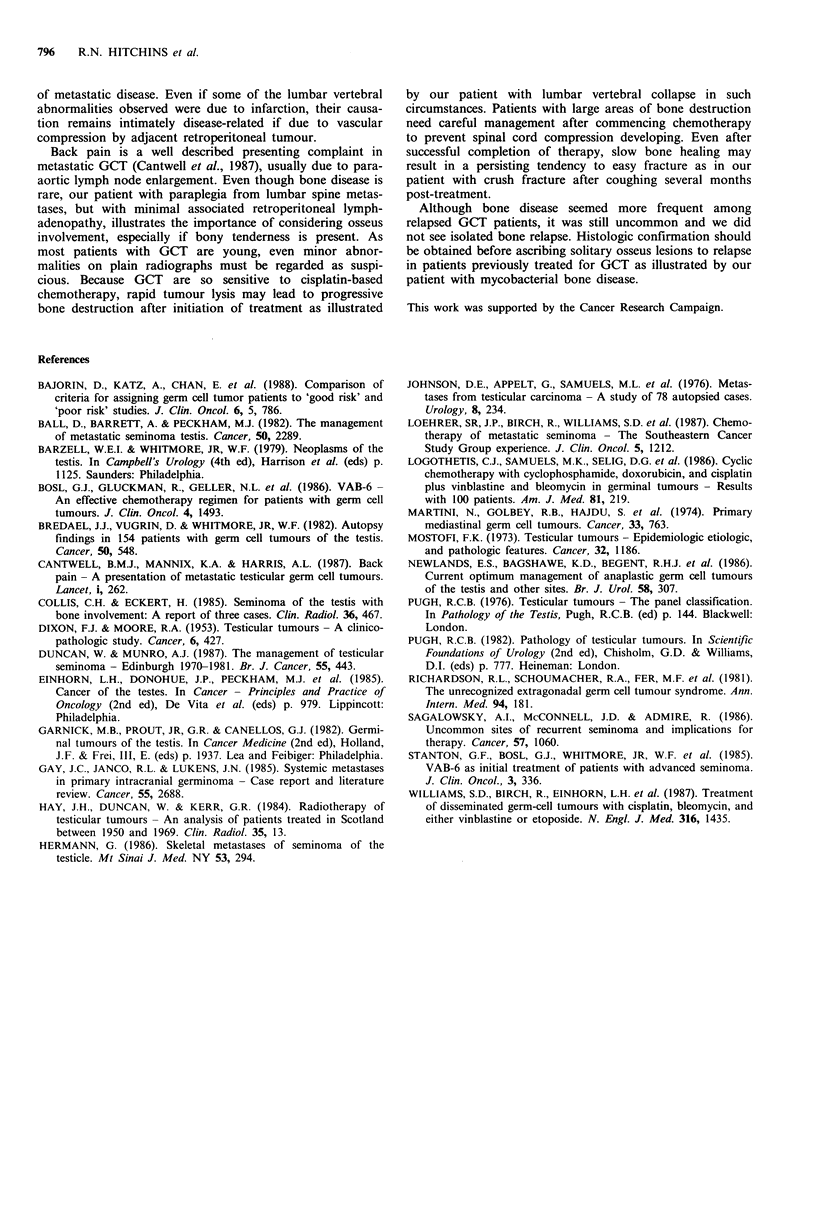# Bone disease in testicular and extragonadal germ cell tumours.

**DOI:** 10.1038/bjc.1988.311

**Published:** 1988-12

**Authors:** R. N. Hitchins, P. A. Philip, B. Wignall, E. S. Newlands, R. H. Begent, G. J. Rustin, K. D. Bagshawe

**Affiliations:** Department of Medical Oncology, Charing Cross Hospital, London, UK.

## Abstract

**Images:**


*Br. J. Cancer* (1988), **58**, 793–796 © The Macmillan Press Ltd., 1988

### Bone disease in testicular and extragonadal germ cell tumours

R.N. Hitchins, P.A. Philip, B. Wignall, E.S. Newlands, R.H.J. Begent, G.J.S. Rustin & K.D. Bagshawe

*Department of Medical Oncology, Charing Cross Hospital, Fulham Palace Road, London W6 8RF, UK.*

**Summary** Of 297 patients with metastatic testicular and extragonadal germ cell tumours (GCT), bone involvement was detected clinically in 3% (7/251) of those at first presentation and in 9% (4/46) of relapsed cases. This difference was not statistically significant (95% confidence limits −2%; +14%). Concurrent systemic metastases, commonly involving lung (7/11 cases) and para-aortic lymph nodes (6/11), were present in all patients with bone disease. All affected patients had localized bone pain and lumbar spine was the most frequent site involved (9/11). Spinal cord compression occurred in two patients while a third developed progressive vertebral collapse after chemotherapy and required extensive surgical reconstruction. At median follow-up of 4 years, survival among patients presenting with bone disease (6/7) was similar to overall survival in the whole group (84%) and appeared better than in those with liver (18/26, 69%) or central nervous system (6/9) metastases at presentation.

Back pain in metastatic germ cell tumours is often due to retroperitoneal lymphadenopathy but lumbar spine osseus metastases must be recognized early if severe potential complications, such as spinal cord compression, are to be avoided. In this series, bone metastases were not seen in the absence of widespread systemic disease suggesting all solitary bony lesions in GCT patients should be biopsied.

Bone is an uncommon site for metastases from testicular and extragonadal germ cell tumours (GCT) (Pugh, 1982). The large study of Dixon & Moore (1953) described metastases seen in 1,000 testicular GCT affecting United States servicemen to January 1948. Autopsy-proven bony metastases were seen in 21% of GCT containing mainly embryonal carcinoma and 36% of those with predominant teratoma but not in other histologic types. Bones of the trunk were involved most commonly. Mostofi (1973) reported 6,000 testicular tumours on the American Registry of Pathology over 25 years and noted a similar distribution of osseus metastases. These pathologic series largely predated the considerable recent advances in treatment of GCT and in methods for demonstrating bone metastases radiologically during life. With optimal radiotherapeutic techniques in seminoma, very high overall cure rates have been possible for over 20 years (Ball *et al.*, 1982; Duncan & Munro, 1987; Hay *et al.*, 1984). Isotope bone scans, computerized tomography (CT) and, most recently, magnetic resonance imaging (MRI) have provided much more sensitive means for diagnosing bone involvement than plain radiographs.

Johnson *et al.* (1976) reported an autopsy series of testicular GCT where 47% of bony metastases were seen among seminoma patients, significantly more than in all other histologic subtypes. Bredael *et al.* (1982) showed similar results in another postmortem series. This apparent change in metastatic pattern from the older series may reflect radiotherapy-induced modification of seminoma natural history with retroperitoneal disease controlled but late recurrence occurring in other sites, including bone, and leading to subsequent death. Now, long term remission and cure in non-seminomatous GCT is common due to widespread use of cisplatin-based combination chemotherapy (Bosl *et al.*, 1986; Logothetis *et al.*, 1986; Newlands *et al.*, 1986; Williams *et al.*, 1987). Such chemotherapy has also proved effective first-line management for bulky metastatic seminoma and as salvage treatment for seminoma patients relapsing after radiotherapy (Loehrer *et al.*, 1987; Stanton *et al.*, 1985).

Bone involvement at presentation with GCT is an adverse prognostic feature equivalent to liver or central nervous system (CNS) metastases according to most authors (Bosl *et al.*, 1986; Logothetis *et al.*, 1986; Williams *et al.*, 1987). However, references to bone secondaries from testicular GCT in recent textbooks of Oncology or Urology are brief (Barzell & Whitmore, 1979; Einhorn *et al.*, 1985; Garnick *et al.*, 1982; Pugh, 1982) and only anecdotal reports of bony metastases from testicular and extragonadal GCT exist (Collis & Eckert, 1985; Gay *et al.*, 1985; Hermann, 1986; Martini *et al.*, 1974; Richardson *et al.*, 1981; Sagalowsky *et al.*, 1986), mostly referring to seminoma. The incidence of clinically detectable osseous metastases, responsiveness of such metastases to currently available chemotherapy, and long term prognosis in patients with bone involvement from GCT remain poorly defined. This paper analyses 10 years' single institution experience with bone metastases from GCT in an attempt to address these issues and describes some unique management problems which arose in affected patients.

Correspondence: E.S. Newlands.
Received 12 May 1988; and in revised form 12 July 1988.

### Patients and methods

Records from 297 male patients treated for metastatic GCT of testicular or extragonadal origin between 1977 and 1987 were examined. Most received first-line chemotherapy for non-seminomatous GCT but some were referred for salvage chemotherapy after relapse and others with advanced seminoma were treated primarily using chemotherapy. Histologic sub-types included seminoma, malignant teratoma undifferentiated (MTU), malignant teratoma intermediate (MTI), malignant teratoma trophoblastic (MTT), and malignant teratoma differentiated (MTD) according to British Testicular Tumour Panel Criteria (Pugh, 1976). All patients underwent regular measurement of serum tumour markers alpha foetoprotein (AFP) and human chorionic gonadotrophin (hCG) as well as serum calcium and alkaline phosphatase. Chemotherapy was administered according to the POMB/ACE (cisplatin, vincristine, methotrexate, bleomycin, actinomycin D, cyclophosphamide, etoposide) protocol (Newlands *et al.*, 1986) or a weekly salvage regimen EP/OMB (etoposide $150\,mg\,m^{-2}$ intravenously (i.v.) plus cisplatin $75\,mg\,m^{-2}$ i.v. alternating with vincristine $1\,mg\,m^{-2}$ i.v., methotrexate $300\,mg\,m^{-2}$ i.v. over 12 h and bleomycin 30 mg i.v. over 48 h).

Bone involvement was diagnosed on the basis of symptoms, usually pain, plus bone destruction demonstrated using one or more imaging technique including plain radiographs, CT scan, isotope bone scan, and magnetic resonance imaging (MRI). Bone biopsy for histologic confirmation was performed in a majority of cases.

### Results

Osseus involvement was noted in 11 cases. Among patients not treated previously, 7 of 251 (3%) had bone disease at presentation and their characteristics are shown in Table I. Most histologic subtypes of GCT were represented in the group with bony involvement. Median follow-up for all non-pretreated patients is 4 years (range one week to 10.8 years) and survival among those with bone involvement (6/7, 86%) was similar to the whole group (84%). By contrast, poorer overall survival was seen among patients with liver metastases at presentation (18/26, 69%) and those with CNS disease at presentation (6/9, 67%). Four of 46 patients (9%) developed bony metastases as part of systemic relapse of their non-seminomatous GCT. Two of these have succumbed despite further treatment.

Coincident metastatic disease elsewhere was noted in all patients with bone involvement, commonly lung (7/11 cases) and para-aortic lymph nodes (6/11). The predominant site of bony involvement was lumbar spine (9/11). Other sites affected were ribs (one case) and skull (two cases) but only two patients had more than one bony site involved concurrently. All patients with bone disease had localized pain. One patient had spastic paraplegia from spinal cord compression at diagnosis, one developed early cord compression after commencing treatment, and a third sustained this complication at relapse. Results of various imaging methods were: plain radiographs showed local bone destruction in 4 of 11 patients, CT scans were positive in 7 of 8 cases with appropriate images obtained, 4 of 6 bone scans performed were positive, and MRI was diagnostic in all three patients who had the investigation performed. Histologic confirmation of osseus metastasis was obtained in 5 cases and changes compatible with necrotic GCT were seen in a sixth patient who had lumbar vertebral biopsy taken at post-chemotherapy retroperitoneal lymphadenectomy. Improvement in the bony changes was evident in two long term survivors who had adequate serial images available. Serum tumour markers were elevated in all but one patient with bone metastases, AFP in 8 of 11 cases and hCG in 8 of 11 cases. The one exception had pure seminoma. Serum alkaline phosphatase was elevated in 7 of 11 cases but raised serum calcium was seen in only one patient. No clinically unrecognized bone metastases were seen among 15 GCT patients who came to autopsy during the period of this study.

Three individual cases are worth highlighting:

*Case I:* A 39 year old man presented with multiple lumbar vertebral metastases, massive para-aortic lymphadenopathy, lung metastases and mediastinal involvement from testicular MTU. He was treated with POMB/ACE chemotherapy (Newlands *et al.*, 1986) and had a rapid response in the tumour but progressive vertebral collapse occurred, threatening spinal cord compression (Figure 1). Extensive surgical reconstruction of the lumbar spine was necessary and tissue obtained at this procedure also revealed MTU. Three months after ceasing the first-line therapy, in apparent complete remission, he sustained an isolated CNS relapse. Owing to the structural problem in the lumbar spine, he had not received CNS prophylaxis with intrathecal methotrexate. The brain metastasis was removed at craniotomy and an Ommaya reservoir inserted. Owing to renal impairment following his prior chemotherapy, he was treated with weekly chemotherapy with carboplatin, etoposide and bleomycin, together with weekly injections of methotrexate in a dose of 12.5 mg into the Ommaya reservoir. He now remains disease-free but needs a walking aid due to residual back problems rather than sequelae of his CNS disease.

*Case II:* A 25 year old male originally presented with painful testicular enlargement which was initially diagnosed as a hydrocoele. While awaiting elective surgery he developed increasingly severe lumbar back pain. Although radiographs were taken of the lumbar spine, the partial collapse of L2 was not noted. Six weeks after seeking medical attention for testicular swelling he developed dense paraplegia due to spinal cord compression and was referred to the Charing Cross Hospital. MRI scan demonstrated the pathology clearly (Figure 2) and interestingly showed para-aortic lymphadenopathy of modest proportions (2–3 cm maximum diameter) at a lower level than the bone lesion. Tissue obtained from the affected vertebra at laminectomy revealed MTU identical to the testicular histology which was obtained concurrently. He has now completed POMB/ACE chemotherapy (Newlands *et al.*, 1986), his tumour is in remission and he has made a partial neurological recovery.

*Case III:* A 57 year old man presented with testicular seminoma causing massive para-aortic lymphadenopathy (>10 cm diameter) and lytic lesions in the lumbar spine on CT scan. Isotope bone scan revealed areas of increased uptake consistent with metastatic disease. The patient received chemotherapy with significant reduction in the para-aortic node mass and then radiotherapy to that region. Serial Ct scans over 8 months since irradiation have shown continued shrinkage of dense residual retroperitoneal tissue but no significant change in the lumbar lytic lesions. Five months after completion of all treatment, the patient complained of acute low back pain after coughing and partial collapse of the fourth lumbar vertebra was noted on plain radiographs. The symptoms resolved over 6 to 8 weeks with supportive treatment.

A twelfth patient, not described so far, also developed a destructive bony lesion in the lumbar spine. He had completed POMB/ACE for a bulky para-aortic and mediastinal metastatic MTI 3 1/2 years previously and presented with back pain and loss of D11–12 intervertebral disc space with bony erosion of D11 vertebra together with an adjacent soft tissue mass. The loss of the intervertebral disc suggested infective aetiology and needle biopsy of the affected area revealed chronic inflammation although a positive culture was never obtained. The patient was treated for an atypical tuberculous infection and was treated with appropriate chemotherapy for 12 months and immobilisation. He is now fully mobile nearly two years after completing his antituberculous therapy.

**Table I** Characteristics of previously untreated GCT patients with bony metastases

| | *Bone involvement* (*n*=7) | *No bone involvement* (*n*=244) |
|---|---|---|
| AGE: median (yrs) | 33 | 29 |
| range (yrs) | 22–57 | 14–61 |
| HISTOLOGY: | | |
| (1) Seminoma | 2 | 24 (10%) |
| (2) MTU | 3 | 119 (49%) |
| (3) MTI | 1 | 62 (25%) |
| (4) MTT | 1 | 23 (9%) |
| (5) MTD | 0 | 8 (3%) |
| (6) Unknown | 0 | 8 |
| PRIMARY SITE: | | |
| (a) Testis | 7 | 216 (89%) |
| (b) Extragonadal | 0 | 28 (11%) |
| OTHER SITES OF DISEASE: | | |
| (i) Para-aortic | 6 | 232 (95%) |
| (ii) Lung | 3 | 77 (32%) |
| (iii) Liver | 0 | 26 (11%) |
| (iv) Mediastinum | 2 | 23 (9%) |
| (v) CNS | 0 | 9 (4%) |

### Discussion

These data suggest clinically detectable bone involvement occurs in 3% of patients presenting with metastatic GCT (95% confidence limits: +1%; +6%). Incidence appeared higher among patients relapsing after previous therapy (9%) but it was not possible to show a statistical difference (95% confidence limits: −2%, +14%). Most of our patients in our series has non-seminomatous GCT so, while our data should reflect incidence of bone metastases in this group accurately, they may be less representative of the seminoma population. None of our affected patients had extragonadal GCT but bone metastases from such tumours are described elsewhere (Gay *et al.*, 1985; Martini *et al.*, 1974; Richardson *et al.*, 1981). Among 58 patients treated for ovarian GCT at this institution over a similar period, no bone involvement was seen. Bones of the trunk, especially lumbar spine, were most commonly affected by metastatic GCT, in accord with historical autopsy series (Dixon & Moore, 1953; Mostofi,

**Figure 1** CT images (a & b) and myelogram films (c) from Case I showing a very large para-aortic lymph node mass with bone destruction of the third lumbar vertebra which onset after commencement of chemotherapy and caused spinal cord compression (myelography dye was introduced from above to show the level).

**Figure 2** (a) Sagittal MR image of Case II, who presented with paraplegia, showing bone destruction and cord compression by tumour mass involving second lumbar vertebra plus para-aortic lymphadenopathy (maximum diameter 2–3 cm) at a lower level (see arrow). Repeat scan (b) was obtained after normalization of tumour markers with chemotherapy.

1973). Longstanding associated radiographic changes reflect the slow rate of bone healing and some of these abnormalities may be permanent.

Survival among our patients presenting with bone disease, was similar to the whole group and appeared better than in those with liver or CNS metastases at presentation. These data suggest osseus metastases may not confer such an adverse prognosis as is commonly believed (Bosl *et al.*, 1986; Einhorn *et al.*, 1985; Logothetis *et al.*, 1986; Williams *et al.*, 1987; Bajorin *et al.*, 1988).

Unfortunately, not all cases of bony involvement described were confirmed histologically. Bone biopsy was undertaken only after careful consideration of potential management implications in each case or if other surgical intervention was necessary. Blood supply to lumbar vertebra may well be compromised by bulky retroperitoneal lymphadenopathy and lead to bone infarction. The possibility that ischaemic necrosis may be the underlying pathology of the collapse of vertebra in some of these patients is the reason we have referred to bone disease in these patients where true bone metastases may not always be present. CT or isotope bone scanning will not distinguish metastatic tumour from bone infarction. However, bone scan abnormalities in all our patients with very bulky retroperitoneal disease did show several 'hot spots' including some in vertebrae not immediately adjacent to the para-aortic node masses, more suggestive of metastatic disease. Even if some of the lumbar vertebral abnormalities observed were due to infarction, their causation remains intimately disease-related if due to vascular compression by adjacent retroperitoneal tumour.

Back pain is a well described presenting complaint in metastatic GCT (Cantwell *et al.*, 1987), usually due to para-aortic lymph node enlargement. Even though bone disease is rare, our patient with paraplegia from lumbar spine metastases, but with minimal associated retroperitoneal lymphadenopathy, illustrates the importance of considering osseus involvement, especially if bony tenderness is present. As most patients with GCT are young, even minor abnormalities on plain radiographs must be regarded as suspicious. Because GCT are so sensitive to cisplatin-based chemotherapy, rapid tumour lysis may lead to progressive bone destruction after initiation of treatment as illustrated by our patient with lumbar vertebral collapse in such circumstances. Patients with large areas of bone destruction need careful management after commencing chemotherapy to prevent spinal cord compression developing. Even after successful completion of therapy, slow bone healing may result in a persisting tendency to easy fracture as in our patient with crush fracture after coughing several months post-treatment.

Although bone disease seemed more frequent among relapsed GCT patients, it was still uncommon and we did not see isolated bone relapse. Histologic confirmation should be obtained before ascribing solitary osseus lesions to relapse in patients previously treated for GCT as illustrated by our patient with mycobacterial bone disease.

This work was supported by the Cancer Research Campaign.

### References

BAJORIN, D., KATZ, A., CHAN, E. *et al.* (1988). Comparison of criteria for assigning germ cell tumor patients to 'good risk' and 'poor risk' studies. *J. Clin. Oncol.* **6**, 5, 786.

BALL, D., BARRETT, A. & PECKHAM, M.J. (1982). The management of metastatic seminoma testis. *Cancer*, **50**, 2289.

BARZELL, W.E.I. & WHITMORE, JR, W.F. (1979). Neoplasms of the testis. In *Campbell's Urology* (4th ed), Harrison *et al.* (eds) p. 1125. Saunders: Philadelphia.

BOSL, G.J., GLUCKMAN, R., GELLER, N.L. *et al.* (1986). VAB-6 – An effective chemotherapy regimen for patients with germ cell tumours. *J. Clin. Oncol.* **4**, 1493.

BREDAEL, J.J., VUGRIN, D. & WHITMORE, JR, W.F. (1982). Autopsy findings in 154 patients with germ cell tumours of the testis. *Cancer*, **50**, 548.

CANTWELL, B.M.J., MANNIX, K.A. & HARRIS, A.L. (1987). Back pain – A presentation of metastatic testicular germ cell tumours. *Lancet*, **i**, 262.

COLLIS, C.H. & ECKERT, H. (1985). Seminoma of the testis with bone involvement: A report of three cases. *Clin. Radiol.* **36**, 467.

DIXON, F.J. & MOORE, R.A. (1953). Testicular tumours – A clinicopathologic study. *Cancer*, **6**, 427.

DUNCAN, W. & MUNRO, A.J. (1987). The management of testicular seminoma – Edinburgh 1970–1981. *Br. J. Cancer*, **55**, 443.

EINHORN, L.H., DONOHUE, J.P., PECKHAM, M.J. *et al.* (1985). Cancer of the testes. In *Cancer – Principles and Practice of Oncology* (2nd ed), De Vita *et al.* (eds) p. 979. Lippincott: Philadelphia.

GARNICK, M.B., PROUT, JR, G.R. & CANELLOS, G.J. (1982). Germinal tumours of the testis. In *Cancer Medicine* (2nd ed), Holland, J.F. & Frei, III, E. (eds) p. 1937. Lea and Feibiger: Philadelphia.

GAY, J.C., JANCO, R.L. & LUKENS, J.N. (1985). Systemic metastases in primary intracranial germinoma – Case report and literature review. *Cancer*, **55**, 2688.

HAY, J.H., DUNCAN, W. & KERR, G.R. (1984). Radiotherapy of testicular tumours – An analysis of patients treated in Scotland between 1950 and 1969. *Clin. Radiol.* **35**, 13.

HERMANN, G. (1986). Skeletal metastases of seminoma of the testicle. *Mt Sinai J. Med.* NY **53**, 294.

JOHNSON, D.E., APPELT, G., SAMUELS, M.L. *et al.* (1976). Metastases from testicular carcinoma – A study of 78 autopsied cases. *Urology*, **8**, 234.

LOEHRER, SR, J.P., BIRCH, R., WILLIAMS, S.D. *et al.* (1987). Chemotherapy of metastatic seminoma – The Southeastern Cancer Study Group experience. *J. Clin. Oncol.* **5**, 1212.

LOGOTHETIS, C.J., SAMUELS, M.K., SELIG, D.G. *et al.* (1986). Cyclic chemotherapy with cyclophosphamide, doxorubicin, and cisplatin plus vinblastine and bleomycin in germinal tumours – Results with 100 patients. *Am. J. Med.* **81**, 219.

MARTINI, N., GOLBEY, R.B., HAJDU, S. *et al.* (1974). Primary mediastinal germ cell tumours. *Cancer*, **33**, 763.

MOSTOFI, F.K. (1973). Testicular tumours – Epidemiologic etiologic, and pathologic features. *Cancer*, **32**, 1186.

NEWLANDS, E.S., BAGSHAWE, K.D., BEGENT, R.H.J. *et al.* (1986). Current optimum management of anaplastic germ cell tumours of the testis and other sites. *Br. J. Urol.* **58**, 307.

PUGH, R.C.B. (1976). Testicular tumours – The panel classification. In *Pathology of the Testis*, Pugh, R.C.B. (ed) p. 144. Blackwell: London.

PUGH, R.C.B. (1982). Pathology of testicular tumours. In *Scientific Foundations of Urology* (2nd ed), Chisholm, G.D. & Williams, D.I. (eds) p. 777. Heineman: London.

RICHARDSON, R.L., SCHOUMACHER, R.A., FER, M.F. *et al.* (1981). The unrecognized extragonadal germ cell tumour syndrome. *Ann. Intern. Med.* **94**, 181.

SAGALOWSKY, A.I., McCONNELL, J.D. & ADMIRE, R. (1986). Uncommon sites of recurrent seminoma and implications for therapy. *Cancer*, **57**, 1060.

STANTON, G.F., BOSL, G.J., WHITMORE, JR, W.F. *et al.* (1985). VAB-6 as initial treatment of patients with advanced seminoma. *J. Clin. Oncol.*, **3**, 336.

WILLIAMS, S.D., BIRCH, R., EINHORN, L.H. *et al.* (1987). Treatment of disseminated germ-cell tumours with cisplatin, bleomycin, and either vinblastine or etoposide. *N. Engl. J. Med.* **316**, 1435.